# Evaluation of methods to quantify aerobic-anaerobic energy contributions during sports and exercise — a systematic review and best-evidence synthesis

**DOI:** 10.3389/fspor.2025.1650741

**Published:** 2025-09-25

**Authors:** Christin Ambaum, Matthias W. Hoppe

**Affiliations:** Exercise Science, Institute of Sport Science and Motology, Philipps University Marburg, Marburg, Germany

**Keywords:** capacity, creatine phosphate, lactate, performance, post-exercise oxygen consumption

## Abstract

**Introduction:**

Energy metabolism during sports and exercise involves both aerobic and anaerobic pathways, with anaerobic contribution playing a key role in various decisive moments during competition. However, unlike the aerobic contribution, quantifying the anaerobic contribution remains challenging due to the lack of a gold standard. This review aims to systematically assess the reliability and validity of different methods to quantify the aerobic-anaerobic energy contributions during sports and exercise, thereby clarifying the level of evidence supporting each method.

**Methods:**

The search was conducted according to the Preferred Reporting Items for Systematic Reviews and Meta-Analyses (PRISMA) 2020 guidelines, including the databases PubMed, Web of Science, Cochrane Library, and BISp-surf on June 11, 2024. Studies quantifying and evaluating the aerobic-anaerobic energy contributions during sports and exercise in humans without diseases, injuries, or disabilities were deemed eligible. Methodological quality was assessed using the COSMIN checklist rating reliability, measurement error, and validity, whereby the overall score was determined using the worst-score-count method. A best-evidence synthesis was also performed to define the direction and level of evidence.

**Results:**

Of the 2,120 studies identified, 34 met the eligibility criteria. Overall, five different methods to quantify aerobic-anaerobic energy contributions during sports and exercise were identified: (i) maximal accumulated oxygen deficit (MAOD), (ii) PCr-La-O_2_, (iii) critical power (CP), (iv) gross efficiency (GE), and (v) the bioenergetic model. Regarding their reliability and validity, the best-evidence synthesis demonstrated that evidence was strong for MAOD and limited to strong for CP and PCr-La-O_2_, and limited to conflicting for GE and the bioenergetic model. Additionally, the validation studies revealed, that the methods differ in terms of their applicability and precision to quantify the anaerobic alactic and lactic contribution.

**Discussion:**

To quantify the aerobic-anaerobic energy contributions during sports and exercise, the MAOD emerged as the most evaluated method and the only one with strong evidence for both reliability and validity. However, as the PCr-La-O_2_ method is the only approach that can distinguish between anaerobic alactic and lactic contributions using direct physiological measures, it should be further evaluated.

## 1 Introduction

Energy metabolism during sports and exercise involves three main pathways: phosphocreatine (PCr) hydrolysis, fast glycolysis with lactate formation, and oxidative phosphorylation of different substrates (1). Their relative contributions are dynamically modulated by exercise intensity and substrate availability (1). Among these pathways, especially the anaerobic energy metabolism plays a key role in various decisive moments during competition: for example, during accelerations and counterattacks in intermittent sports, as well as breakaways and final sprints during endurance disciplines (2, 3). Despite its significance, quantification of the anaerobic contribution remains challenging. Unlike the aerobic contribution, which can be validly assessed by oxygen (O_2_) uptake and respiratory gas analyzers, anaerobic contribution lacks a universally accepted gold standard (4). Consequently, multiple approaches have been proposed to estimate anaerobic contribution across different exercise modalities, resulting in the development of various methods and methodological frameworks. However, since the aerobic and anaerobic energy systems are intricately interconnected, knowledge of both systems is necessary (5). With respect to the anaerobic energy contribution during sports and exercise, five different methods were frequently investigated.

The first method is the maximal accumulated oxygen deficit (MAOD). It is based on the principle that, during high-intensity exercise exceeding maximal oxygen uptake, the total energy demand surpasses the capacity of aerobic supply, necessitating anaerobic energy supply (6). Since there is a linear relationship between power output and oxygen uptake, the MAOD can be determined by subtracting the total measured oxygen uptake over the course of supramaximal exercise from the estimated accumulated oxygen demand (6). As a result, MAOD quantifies the difference between the estimated total oxygen demand and the actual oxygen uptake, reflecting the energy provided by anaerobic metabolic pathways (7).

The second method is the PCr-La-O₂ method. Contrary to MAOD, it describes the energy supply as the sum of three components: PCr breakdown, fast glycolysis, and oxidative phosphorylation (8). This method is fundamentally linked to the excess post-exercise oxygen consumption (EPOC), particularly the fast component (EPOC_fast_) (9, 10). Since the PCr-La-O₂ method accounts for PCr as a primary anaerobic energy source, it directly corresponds to EPOC_fast_, which is dominated by the replenishment of PCr and restoration of oxygen stores, requiring increased post-exercise oxygen uptake (11). Therefore, only the PCr-La-O₂ method allows for the distinction between anaerobic alactic and lactic energy contributions (12).

A third method is the critical power (CP) (13). It represents the highest sustainable power output that can be maintained over an extended time period and at which adenosine triphosphate (ATP) resynthesis is predominantly supported by oxidative phosphorylation (14). Below CP, oxygen uptake reaches a plateau, where ATP resynthesis is primarily driven aerobic. Contrary, exceeding CP leads to an increased reliance on fast glycolysis, accelerating muscular glycogen depletion and accumulation of lactate (15). Thus, the curvilinear power-time relationship used to define CP provides an estimate of the finite anaerobic work capacity (W'), reflecting the energy produced by PCr hydrolysis, fast glycolysis, and myoglobin oxygen stores (16). Therefore, the capacity to perform work above CP is limited.

The fourth method is the gross efficiency (GE). It allows to quantify the mechanical efficiency of muscular work during exercise, particularly during cycling. It is defined as the ratio of mechanical power output to metabolic power input (17). The power input can be calculated from the oxygen uptake and its equivalent. The aerobic power can be calculated from the metabolic power input and efficiency at which metabolic power is converted to mechanical power (16). Subsequently, the anaerobic mechanical power can be calculated by subtracting the aerobically ascribable mechanical power from the total power output produced.

The last approach is the bioenergetic model, which mathematically represents the contribution and interaction of the aerobic, lactic, and alactic metabolic pathways during exercise based on changing intensity and duration (18, 19). Using a hydraulic tank analogy, each energy system is modeled as a reservoir with specific capacities and flow rates. Aerobic metabolism responds more slowly but is sustained, while lactic and alactic systems react rapidly with limited capacity. Governed by differential equations, the model simulates energy system dynamics from oxygen uptake and power output data, allowing individualized estimation under variable-intensity conditions (18, 19).

Since the quantification of aerobic-anaerobic energy contributions is based on the methods used (16), the results completely underly its determinations. Consequently, it is essential to consider their reliability and validity. Taking this and the five described methods to estimate the energy contribution during sports and exercise into account, previous research has either investigated the reliability of one method or compared two methods in terms of their validity (20–24). Since the methods were introduced across different decades and have been modified to varying extents (4), there are disparities in the number of application- and evaluation-based studies. Preliminarily, based on the available studies, but without scientific evidence, MAOD seems to be the most commonly used and studied method in the field. With regard to overview studies, a limited number of narrative reviews have examined MAOD and CP in terms of their influencing factor and practical applications (4, 16, 25–27). Moreover, there is only one narrative review, discussing the advantages, limitations, and practical applications of MAOD, CP, and GE (16). Unfortunately, this review did not consider the PCr-La-O₂ method. While the narrative reviews provide detailed background information about the underlying energy metabolism (4, 16, 25–27), there is, to the best of our knowledge, no systematic review that highlights the evaluation and extracts the reliability and validity of the different methods yet.

Therefore, this systematic review aims to assess the reliability and validity of different methods to quantify the aerobic-anaerobic energy contributions during sports and exercise, thereby clarifying the level of evidence for each method.

## 2 Methods

### 2.1 Search strategy

The systematic review was conducted according to the Preferred Reporting Items for Systematic Reviews and Meta-Analyses (PRISMA) 2020 guidelines (28). The literature search included the databases PubMed, Web of Science, Cochrane Library, and BISp-surf and was completed on June 11, 2024. The PICO (P = Population, I = Intervention, C = Comparison, O = Outcome) scheme (28) was used to develop a search strategy: P = everyone who is suitable for sports and exercise, except for patients with diseases, injuries, or disabilities; I = methods to quantify aerobic-anaerobic energy contributions during sports and exercise; C = evaluation, reliability, or validity; O = proportion of aerobic-anaerobic supply. However, the component for Population (P) was excluded from the search term to make sure that all type of athletes were included. The subsequent search term was applied to all databases with no restrictions: (component model OR maximal accumulated oxygen deficit OR MAOD OR critical power OR CP OR gross efficiency OR GE OR metabolic power model OR Pmet OR VLamax OR PCr-La-O_2_ OR muscle biopsy OR MRI OR fast component OR EPOC fast) AND (sports OR exercise OR test) AND (evaluation OR reliability OR validity OR comparison OR relationship) AND (anaerobic). All results were converted into a citation manager (Clarivate Analytics, EndNote X9.2, London, UK) and transferred to a spreadsheet (Microsoft Office, Excel 2021, Redmond, USA). After duplicates were removed, titles, abstracts, and full texts were screened for eligibility criteria. Studies that were considered to be unfitting were eliminated. In addition, supplementary search was performed by reviewing the reference lists of the studies considered eligible. All methodological procedures were completed independently by two researchers. When disagreement arose, consensus was reached through discussion or the decision of a third researcher.

### 2.2 Eligibility criteria

To be included, the studies had to meet the eligibility criteria that were specified and agreed by both authors. The following criteria for screening titles and abstracts were:

- Written in English
- No systematic review or book section
- No patients, injured, disabled or animals, plans, microbiomes, and in vitro experiments
- Topic on energy contribution during sports and exercise

The criteria for full texts were as follows:

- Full text found
- Original study
- Quantification of aerobic-anaerobic energy contributions
- Evaluation of a method to quantify the former

### 2.3 Assessment of methodological quality

The methodological quality of the studies to investigate the reliability and validity was implemented using the Consensus-based Standards for the selection of health Measurement Instruments (COSMIN) checklist (29, 30) as recommended by Ma et al. (31). Of the checklist, boxes 6–9a were used for reliability, measurement error, criterion validity, and convergent validity, respectively. Each item was rated as 3 = very good; 2 = adequate; 1 = doubtful; 0 = inadequate; NA = not applicable. The overall quality and risk of bias of each study was subsequently rated based on the worst-score-count method, meaning that the lowest scoring item was decisive for the overall score (30).

### 2.4 Data extraction

Content of all included studies was summarized using the PICO scheme. Extracted information concerned (if applicable): P = number of participants, age, sex, type of sport, level; I = information about the setting of the study; C = description of used methods to quantify aerobic-anaerobic energy contributions; O = main results.

The mean differences and corresponding effect sizes (ES) according to Cohen's d were extracted directly from the studies, if available. Effect sizes were classified according to Cohen (32): trivial (<0.2), small (0.2 to <0.5), moderate (0.5 to <0.8), and large (≥0.8). For reliability and validity assessments, intraclass correlation coefficients (ICC), Pearson's correlation coefficient (r), and the coefficient of variation (CV) or typical error (TE) were considered. The magnitude of correlations was classified as (32): very small (<0.1), small (0.1 to <0.3), moderate (0.3 to <0.5), and large (≥0.5). ICC was classified accordingly: poor (<0.5), moderate (0.5 to <0.75), good (0.75 to <0.9), and excellent (≥0.9) (33). The CV values were interpreted as excellent (≤10%), good (10 to <20%), acceptable (20 to <30%), and poor (≥30%) (34).

Due to the heterogeneity of the included studies regarding the applied methods and their calculations, a meta-analysis was not possible to perform. Alternatively, a best-evidence synthesis was made to clarify the direction and level of evidence of the different methods. Therefore, the criteria according to Asker et al. (35) were used to set evidence as strong, moderate, limited, or conflicting (Table 1).

**Table 1:** Criteria for the best-evidence synthesis according to Asker et al. (35).

Rating	Study quality	Criterion
Strong evidence	≥2 high-quality studies	≥75% consistent findings on these studies
Moderate evidence	1 high-quality study and/or ≥2 moderate quality studies	≥75% consistent findings in these studies
Limited evidence	1 moderate quality study and/or ≥1 low-quality studies	–
Conflicting evidence	≥2 studies of any quality	<75% consistent findings in these studies

## 3 Results

**Figure 1:**
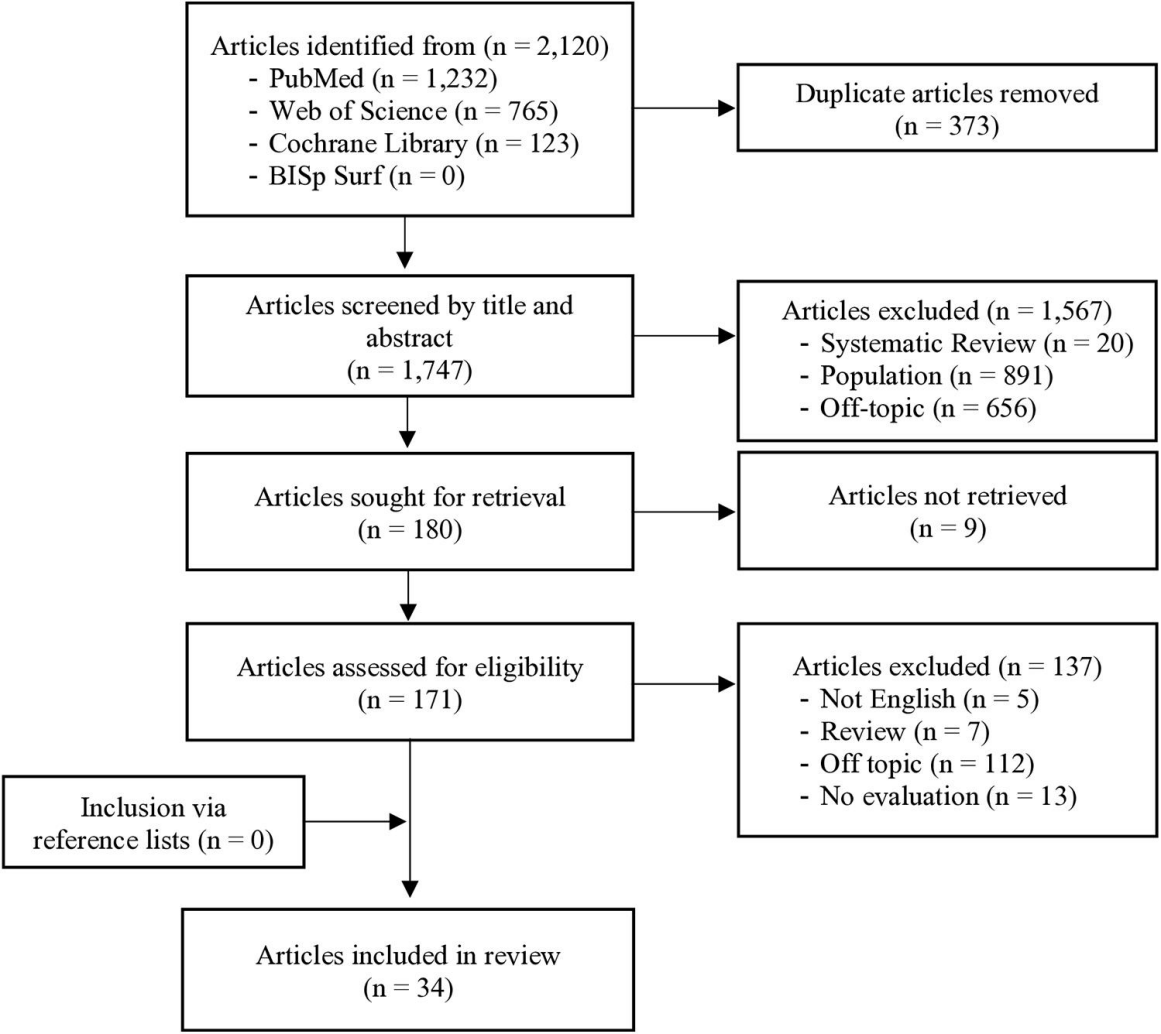
Flowchart of the literature search including the study selection process according to the PRISMA guidelines.

A total of 2,120 studies were identified. After removing 373 duplicates, 1,747 articles were screened for titles and abstracts, whereby 1,567 did not meet the eligibility criteria. Of the remaining 180 full texts, 47 fulfilled the criteria. After excluding 13 application studies (22, 36–47), 34 studies were finally included. No additional studies were identified through screening the reference lists. The most common reason for the exclusion was an unsuitable study population (*n* = 891), followed by an unrelated topic to energy contribution during sports and exercise (*n* = 656), and the missing quantification of aerobic-anaerobic energy contributions (*n* = 112). Figure 1 shows the detailed selection process.

### 3.1 Study characteristics

Table 2 gives an overview of the study characteristics. Of the 34 studies included, five different methods to quantify aerobic-anaerobic energy contributions during sports and exercises were detected, namely: (i) MAOD, (ii) PCr-La-O_2_ method, (iii) CP, and (iv) GE. Less investigated was an identified fifth method, the so called (v) bioenergetic model.

**Table 2:** Study characteristics and results of the included studies using the PICO scheme.

Study (Year)	Population	Intervention	Comparison	Outcome
Andersson and McGawley (20)	21 junior cross-country skiers (11 males, 10 females) (18 ± 1 years) at national or international level	4 × 4 min continuous, submaximal roller-skiing at 5.2–10.0 km/h and 7° incline on a treadmill with increasing speed of 0.8–1.0 km/h every minute, followed by a 1 min break; 1 incremental test until exhaustion, starting at 10–12 km/h and 3–4° incline with increasing speed by 0.4 km/h every minute and increasing incline by 1° every minute up to a maximum of 9°, followed by a 2.5 h break; 1 600 m self-paced time trial (TT)	Comparison of the anaerobic contribution determined by four different models with the MOAD (4 + Y, 4-Y), gross efficiency (GE) and submaximal energy cost (EC) during a continuous cross-country roller-skiing protocol	Application of the GE and EC method resulted in identical estimations of oxygen deficit; oxygen deficit was significantly lower with 4 + Y compared to 4-Y and GE/EC (*p* < 0.05; ES = 0.64); mean difference between the oxygen deficit estimated with the 4 + Y vs. 4-Y method was −6.3 ± 4.9 ml/kg, with the 4 + Y vs. GE/EC method was −7.2 ± 1.2 ml/kg and with the 4-Y vs. GE/EC method was −1.0 ± 5.3 ml/kg, with respective TE of 5.3% (3.5 ml/kg), 1.9% (0.8 ml/kg), and 6.0% (3.8 ml/kg); oxygen deficits estimated with the 4 + Y vs. GE/EC method were highly correlated (r = 0.99; *p* < 0.05)
Andersson et al. (57)	15 endurance-trained athletes (8 males, 7 females) (31 ± 7 years)	4 × 5 min submaximal, continuous treadmill runs with different intensities between 55 and 80% of VO_2_peak (9.7–13.2 km/h) with increasing velocity, followed by 10 min rest and 1 × 4 min time trial beginning at the last submaximal stage minus 2 km/h	Comparison of estimated anaerobic energy contribution with four different models: two linear models 5 + Y_LIN_ (with a baseline metabolic rate) 5-Y_LIN_ (without), GEC_AVG_ (average over all stages) and GEC_LAST_ (last stage only), all using the integration of the metabolic rate over the 4-min time trial	The estimated anaerobic contribution was significantly lower for the 5 + Y_LIN_ method compared to the three other models (5-Y_LIN,_ GEC_AVG_, GEC_LAST_ (∼26%; *p* = 0.002); there were high TE for the respective comparison except for the 5 + Y_LIN_ vs. GEC_AVG_ model (TE = 0.03)
Andrade et al. (48)	14 male runners (36 ± 2 years)	First day: 1 maximal incremental test on the treadmill; second day: 1 7 min run at 50% of VO_2_max, one supramaximal run at 110% of VO_2_max until volitional exhaustion and 1 7 min run at 70% VO_2_max, all interspersed by 25–35 min rest; third to seventh day: performance of 5 bouts between 55 and 95% of VO_2_max and one supramaximal bout at 110% VO_2_max	Comparison of the anaerobic contribution determined by the conventional MAOD method and by the backward extrapolation technique for different submaximal running intensities	Low ICCs and high TE and CVs for absolute (ICC = 0.26; TE = 2.03; CV = 46.2%) and relative (ICC = 0.24; TE = 24.9; CV = 47.5%) MAOD values; strong correlation between conventional MAOD and backward extrapolation for absolute (r = 0.86) and relative (r = 0.85) MAOD; no significant differences were found between the conventional MAOD values and backward technique values (*p* > 0.05); Low coefficients of determination for the backward extrapolation (r^2^ = 0.60)
Bangsbo et al. (61)	8 physically active males (23–29 years)	One-legged, dynamic knee-extensor exercises on an ergometer with workloads at 10 W (for 10 min); one constant-load test at 65 W until exhaustion, followed by a recovery period of 1 h and a final incremental exercise test with 10–50 W with each step lasting 7–8 min	Comparison of the anaerobic contribution via muscle biopsies (M. quadriceps) and oxygen deficit method for the leg and whole-body during knee-extensor exercises at different intensities	The anaerobic contribution estimated from muscle biopsy relates extremely well in quantity to the estimated oxygen deficit (91.2 vs. 91.6 mmol ATP/kg wet weight)
Bergstrom et al. (70)	9 recreationally trained subjects in cycling (*n* = 2) or running (*n* = 8) (4 males, 5 females) (23 ± 3 years)	1 incremental test on a cycle ergometer at 70 rpm with increasing intensity of 30 W every 2 min until exhaustion; 4 randomly ordered constant power tests at 70–105% of VO_2_peak and a 3 min all-out test on a cycle ergometer	Comparison of CP and anaerobic work capacity (W’) estimated by 5 different mathematical models: linear-TW, linear-P, nonlinear-2, nonlinear-3 and CP_3min_ model	The 5 estimates for W’ showed significant mean differences (*η*^2^ = 0.525; *p* < 0.001); nonlinear-3 (15.2 ± 5.6 kJ) and nonlinear-2 models (14.6 ± 5.5 kJ) produced significantly higher estimates of W’ than the linear-TW (12.2 ± 5.8 kJ), linear-P (11.4 ± 6.1 kJ) and CP_3min_ (10.4 ± 2.6 kJ) models (*p* < 0.05)
Bosquet et al. (21)	17 middle- and long-distance runners (23 ± 3 years)	1 incremental test on a treadmill with initial speed set at 2.8 m/s and increments of 0.28 m/s every 2 min until exhaustion; 5 constant velocity tests at 95–120% of peak treadmill velocity until exhaustion, randomly ordered; 1 800 m time-trial on an indoor track	Comparison of anaerobic running capacity (ARC) estimated from four different methods of Hill, Monod and Scherrer, Whipp and Morton during constant velocity tests and 800 m time-trial in running	ICC for all ARC estimations was 0.52; ARC was moderately correlated with oxygen deficit (*r* = 0.49–0.57), except for the method of Hill; ARC determined from Morton was significantly higher than ARC derived from Whipp or Hill (ES = 2.52–2.76; *p* < 0.001) and moderately correlated (r = 0.65–0.75; *p* < 0.05); ARC computed from Morton was significantly higher compared to the oxygen deficit (ES = 1.99; *p* < 0.01)
Bosquet et al. (49)	19 moderately to highly trained middle-and long-distance runners (23 ± 3 years)	1 maximal graded exercise test on a treadmill with increasing speed of 1 km/h every 2 min until exhaustion, followed by 6 randomly ordered constant-speed tests of 95–120% peak treadmill speed, separated by 72 h	Comparison of MAOD estimated from three different methods of Medbø (1988), Whipp (1986) and Hill (1998) during treadmill running	There was no difference between MAOD values from Medbø and Hill, they were not associated and showed wide limits of agreement (LoA=±0.038 ml/min/kg; r = 0.25; *p* > 0.05); the method of Whipp showed largely lower estimations for MAOD than the other (LoA =± 35.6 and ± 23.8 ml/kg; ES > 1.94; *p* < 0.001); correlations show no association between MAOD from Hill with other estimates (r = 0.21–0.33; *p* > 0.05) and no relationship between Whipp and Medbø (r = 0.33; *p* > 0.05)
Buck and McNaughton (62)	8 trained male cyclists (25 ± 7 years)	1 incremental test on a cycle ergometer with resistance increasing 25 W/min; 10 submaximal bouts of 10 min between 30 and 90% of VO_2_max; 1 supramaximal test at 110% of VO_2_max until cadence was <80 rpm or volitional exhaustion	Comparison of MAOD using 2–10-point regressions and evaluation of the effect of the number of submaximal exercise bouts	Sequential removal of the highest or lowest submaximal bouts resulted in progressively larger differences in MAOD compared to the 10-point regression (24.7 vs. 67.4 ml O_2_ eq/kg); removing the most central bouts led to a significant smaller MAOD compared to the other methods
Campos et al. (58)	6 swimmers (3 males, 3 females) (15 ± 2 years)	3 experimental swimming sessions, interspersed by 24 h; (1) 4 submaximal efforts (>5 min); (2) 1 submaximal bout, followed by a maximal 400 m front crawl; (3) 1 maximal bout 400 m front crawl	Comparison of three determinations of accumulated oxygen deficit: AOD, AC_ALT_ (measured continuously with a snorkel) and AC_FS_ (measured without a snorkel) during 400 m maximal swimming efforts	Relative AOD, AC_ALT_ and AC_FS_ values showed significant differences (*p* = 0.04), post-hoc analysis indicated no differences; AOD was highly correlated with AC_ALT_ (r = 0.95; *p* = 0.002) and AC_FS_ (r = 0.82; *p* = 0.04); LoA of AOD and AC_ALT_ were 0.96 and 0.87 L; LoA between AOD and AC_FS_ were 0.77 for upper limit and 2.26 L for lower limit
Doherty et al. (50)	15 physically active male sports students (22 ± 3 years)	3 × 6 min treadmill runs of increasing intensity at 10.5% incline, interspersed by 5 min rest, followed by one incremental treadmill test with increasing velocity 0.14 m/s every minute until exhaustion; 1 supramaximal treadmill test with 6 × 15 s running bouts at 125% VO_2_max with 15 s rest in between	Assessment of the reliability of MAOD during supramaximal running at 125% compared to the extrapolation method of Medbø (1988)	ICC was excellent (0.91) and CVs were 6.8% for MAOD; 95% LoA for MAOD were ± 15.1 ml O_2_ eq/kg; no systematic bias for MAOD (*p* = 0.51); correlation between absolute MAOD residual errors and scores was r = −0.14; *p* = 0.38
Ebreo et al. (75)	13 males (35 ± 5 years), 2 females (25 ± 5 years) with a minimum of 6 h training/week	1 maximal incremental exercise test on a cycle ergometer (15 W/s) until volitional exhaustion or cadence <60 rpm; 2 high intensity exercise tests (P1, P2) with 6 min at 50% MAP (Pre), 2 min 25 W, 4 min 80% or 100% MAP, 1 min 25 W and 10 min 50% MAP (Post)	Comparison of GE during high intensity exercise using the back-extrapolation method (BGE) or the conventional submaximal method (GE) to assess the reliability and validity	CVs were 7.8% (P1) and 9.8% (P2) in BGE; LoA were ±3.6% vs. ± 3.74% (P1) and ±4.2% vs. ± 4.1% (P2) for GE vs. BGE; CVs for anaerobic contribution were 3.5% vs. 2.9% (P1) and 6.8% vs. 5.0% (P2) for GE vs. BGE; high correlations of BGE and GE in P1 Post (r = 0.98; *p* = 0.01) and in P2 Post (r = 0.80; *p* = 0.01); no significant correlations between BGE and GE Pre in P1 (21.1% vs. 20.9%, *p* = 0.29)
Gaesser et al. (71)	16 physically active males (21 ± 1 years)	Maximal incremental cycling test with increasing intensity of 30 W/min until volitional fatigue; 5–7 constant-load exercise bouts until exhaustion on a cycle ergometer of sub- and supramaximal peak power attained during the first test	Comparison of AWC from 5 different CP models [three-parameter nonlinear, two-parameter nonlinear, linear (P x t), linear (P), exponential] during cycling with different exercise intensity and duration	AWC estimates differed significantly between the five models; the three-parameter model provided the highest AWC, the linear (P) model the lowest (58 ± 19 kJ vs. 18 ± 5 kJ); goodness of fit was significantly lower for the linear (P) model compared to all others (R^2^ = 0.96 ± 0.03; *p* = 0.005); correlations for AWC between the two linear models and the two-parameter nonlinear model were high (r = 0.95–0.99; *p* < 0.001); none of the correlations for the three-parameter nonlinear model were high (r = 0.25–0.64; *p* > 0.05-<0.05)
Hatauta et al. (67)	7 sprinters (23 ± 0 years) 7 middle-distance runners (21 ± 2 years) with a minimum of 5 training sessions/week	1 Submaximal cycling test with 5 stages between 80 and 140 W, each lasting 4 min, interspersed by 2 min rest; 1 graded exercise test with 30 W/min increase until cadence was <85 rpm for 10 s; 1 supramaximal exercise bout at 115% VO_2_max until volitional exhaustion	Comparison of anaerobic contributions derived from PCr-La-O_2_ and MAOD in sprinters and middle-distance runners	No significant correlation between PCr-La-O_2_ and MAOD method (r = −0.06; *p* > 0.05); no difference between the calculated anaerobic contribution from PCr-La-O_2_ and MAOD method (44.6 ± 3.0 vs. 45.2 ± 5.1%, *p* = 0.79); significantly higher values for Sprinters in energetics from glycolytic pathway and blood lactate concentration (*p* = 0.02)
Hill and Smith (73)	Physical education students 13 males (23 ± 2 years) 13 females (23 ± 2 years)	5 all-out exercise bouts on a cycle ergometer until exhaustion with power outputs between 3.5–6.5 W/kg for females and 4.0–8.5 W/kg for males	Comparison of anaerobic contribution from a linear power-time relationship (critical power) and MAOD	Strong correlation between the linear power-time relationship and MAOD (r = 0.77; *p* < 0.01); no significant difference between the two determinations of anaerobic contribution (*p* = 0.44)
Hill (72)	5 males (21 ± 1 years) 5 females (21 ± 2 years), recreationally active in sports or fitness activities	2 predicting trials 5 exhaustive cycling tests with individually selected power outputs, lasting ∼3–10 min with ∼80 rpm until the cadence fell <60 rpm; all tests were separated by at least 48 h	Comparison of anaerobic contribution derived from 3 different critical power models (2-parameter model, 3-parameter hyperbolic model, 3-parameter exponential model) in cycling	CP was largest from the 3-parameter exponential model (209 ± 51 W) and significantly different between all three models (*p* = 0.003); anaerobic contribution was significantly higher when derived from the 3-parrameter hyperbolic model when compared to the 2-parameter hyperbolic model (25.3 ± 13.2 vs. 20.4 ± 9.0 kJ; *p* = 0.048); SEE for the 2-parameter hyperbolic model was significantly lower compared to the 3-parameter hyperbolic model (1.0 ± 1.0 vs. 12.4 ± 15.2 kJ; *p* = 0.049)
Hill et al. (68)	17 males (23 ± 3 years) 13 females (22 ± 2 years), recreationally active in sports	1 incremental treadmill test with 2 min stages from 135 to 165 m/min; 3 randomized constant-speed tests at 92% of peak speed, lasting 3 min, 7 min or until exhaustion	Comparison of anaerobic contribution from PCr-La-O_2_ method and oxygen deficit in running	Highly significant correlations between PCr-La-O_2_ and oxygen deficit method (r = 0.80–0.94; *p* < 0.01), highly significant correlation between the two methods across the three durations (r = 0.99; *p* = 0.001); ES for the differences between methods were 0.32, 0.36 and 0.52 for the 3 min, 7 min and exhaustive test, respectively; significant effect of method (*p* < 0.001) and duration (*p* < 0.001) but no significant interaction effect
Kalva-Filho et al. (51)	4 male (19 ± 1 years) 5 female (18 ± 2 years) swimmers at regional and national level	2 incremental swimming tests starting at 20N and increasing 10N every 3 min; 6 randomized, 7 min submaximal swimming tests at intensities ranging from 50 to 90% of maximal aerobic force; 2 maximal swimming tests at 100% of maximal aerobic force until volitional exhaustion	Test-retest reliability of MAOD in submaximal and maximal tethered swimming	Significantly high ICCs for MAOD Test-Retest during maximal effort (ICC = 0.89–0.93; *p* < 0.05); CVs (9.5–9.6%) und TE (4.3%) were low; MAOD values did not differ significantly between the tests (*p* > 0.87)
Kaufmann et al. (12)	16 male state-level handball players (23 ± 3 years)	30–15 intermittent running test until exhaustion, performed twice within 2 weeks	Test-retest reliability of the conventional PCr-La-O_2_ and intermittent PCr-La-O_2int_ during intermittent running	Estimates for aerobic share showed smallest limits of agreement for both methods [CV%: 3.62 and 6.06 (int)]; limits of agreement for anaerobic lactic share were CV%: 14.85 and 9.98 (int) and for anaerobic lactic CV%: 11.43; limits of agreement for overall anaerobic share were CV%: 7.49 and 8.95 (int)
Lidar et al. (18)	11 male cross-country skiers (24 ± 4 years) at national and international levels	4 submaximal exercise tests; 2 self-paced roller-skiing sprint time trials (STT) on a treadmill, consisting of 3 flat sections (1°) and 2 uphill sections (7°) resulting in a course of ∼1,280 m; both trials interspersed by 45 min of recovery	Comparison of four bioenergetic models (2TM-fixed, 2TM-free, 3TM-fixed and 3TM-free) estimating the aerobic and anaerobic contribution during sprint roller-skiing	The model-to-measurement mean difference (0.5) and TE for the anaerobic contribution were lower but not significant for the 2TM-free compared to the other models (TE = 0.6; *p* = 0.103); the RMSE of the anaerobic contribution were the lowest for the 2TM-free and the highest for the 3TM-fixed model (11.7% vs. 17.2%; 50.0–77.6 W vs. 104.1–106.1 W); the relative energy contribution from the alactic system and the lactic system to the total anaerobic contribution was 38.6% and 61.4% for the 3TM-free, and 38.7% and 61.3%, for the 3TM-fixed model
Lidar et al. (19)	14 well-trained, male cyclists (35 ± 8 years)	1 submaximal incremental cycling test with initial load of 80W, increased by 20 W/3 min until RQ > 1.0 (P1a); 1 maximal incremental cycling test with initial load of 100 W, increased by 40 W/min until exhaustion or cadence <70 rpm (P1b); 2 intermittent protocols with various and individualized power outputs on two different days (P2 and P3)	Comparison of the measured and modelled metabolic energy supply during different cycling protocols	SD of the average RMSE was 38.5% (P3); LoA for measured and modelled data for aerobic metabolic rate were −2.75 W (−124.80–119.29 W) for P2 and −6.73 W (−148.76–135.30 W) for P3; mean absolute percentage error was 8.6 ± 1.5% for P2; there were significant differences between modelled and measured data for the aerobic and anaerobic contribution at several stages during the intermittent protocol (*p* ≤ 0.001–0.036)
Luches-Pereira et al. (66)	12 males (26 ± 3) physically active	1 graded incremental exercise test with 13W/min on a one-legged knee-extensor ergometer until volitional exhaustion; 2 constant-load exercise tests at 100% (TTF100) and 110% (TTF110) of peak power output on a knee-extensor ergometer until exhaustion; performed twice and separated by ≥ 24h	Assessment of the test-retest reliability for PCr-La-O_2_ method in maximal and supramaximal knee-extensor exercises	TTF100: ICC was moderate and significant (0.71, *p* = 0.004); CVs were between 6.0% and 37.8%; LoA were between −591.7 and 753.5 ml O_2_; SEM was 240.1 ml O_2_; no significant differences between PCr-La-O_2_ values (*p* > 0.111); TTF110: ICC was moderate and not significant (0.44, *p* = 0.085); CVs were between 3.3 and 60.4%; LoA were between −1,188.and 1,002.4 ml O_2_; SEM was 389.6 ml O_2_;no significant differences between the repeated trials for any of the studied values (*p* > 0.086), among others: aerobic (*p* = 0.439), alactic (*p* = 0.356) and lactic (*p* = 0.242) shares; significant difference between the anaerobic contribution at TTF100 and TTF110 (*p* = 0.042)
Maturana et al. (69)	9 males and 4 females (26 ± 3 years), recreationally or competitively active in cycling at a provincial level	One incremental ramp test on a cycle ergometer, starting at 50 W for 4 min, followed by increments of 30 W/min for males and 25 W/min for females 5 constant-power output trials to exhaustion on a cycle ergometer at ∼70–110% of peak power output with a cadence of 70–105 rpm, lasting ∼1–20 min and assigned randomly	Comparison of CP and W’ estimated by five mathematical models (CP_exp_, CP_3−hyp_, CP_2−hyp_, CP_linear_, and CP_1/time_) (and different numbers of TTE trials 1,2,3,4,5) during cycling. CP_3−hyp_ is used as the criterion method	CCC was good to excellent (0.78–0.99) for all models and time trials; the model that predicted data most accurately was confirmed as the CP_3−hyp(1,2,3,4,5)_ (R^2^ = 0.99; RMSE = 26.5 W); RMSE ranged from 2.44–22.90 W and was lowest for CP_linear (2,3,4,5)_ and highest for CP_1/time (1,2)_; for the methods CP_2−hyp(1,2,3)_, CP_linear(1,2)_, CP_linear(1,2,3)_, CP_1/time(1,2,3)_, CP_1/time(1,2,3,4)_, and CP_1/time(1,2,3,4,5)_ the difference in relation to the criterion method was considered likely positive (overestimation); the methods CP_3−hyp(1,2,3,4)_, CP_3−hyp(2,3,4,5)_, CP_2−hyp(3,4,5)_, CP_2−hyp(2,3,4,5)_, CP_2−hyp(1,2,3,4,5)_ as well as CP_linear_ and CP_1/time_ using the trials (3,4), (4,5), and (3,4,5) resulted in a very small chance of underestimating W’; the inclusion of trials lasting <10 min (trials 1–3) caused a substantial underestimation of W’
Medbø and Tabata (63)	16 male students (25 ± 1 years)	9 submaximal tests at 30–90% of VO_2_max on a cycle ergometer; 3 supramaximal cycling bouts lasting 30 s (8.9 ± 0.2 W/kg), 1 min (6.4 ± 0.2 W/kg) or 2–3 min (4.8 ± 0.2 W/kg) until exhaustion	Comparison of anaerobic energy contribution derived from muscle biopsies of M. vastus lateralis or accumulated oxygen deficit during cycling bouts lasting 30s-3min	High correlation of ATP turnover rate for the whole body determined from oxygen deficit or calculated from muscle biopsies (r = 0.94); the amount of anaerobic energy release was 32% less for 30 s than for exercises lasting ≥1 min (*p* < 0.03); lactate production accounted for >75% of the anaerobic ATP production
Medbø and Welde (65)	13 moderately trained participants (10 males, 3 females)	12 subjects performed 10–15 bouts of 10 min continuous cycling at 90 or 45 rpm from intensities with zero loads up to 95% of VO_2_max; 9 subjects performed an incremental test with 4 min stage duration and increase of 22 W/stage (11W for females) at cadences of 90 and 45 rpm; 9 subjects cycled with zero load and 30 rpm for 10 min	Comparison of 8 different calculations (M1-M8) of the MAOD using different intercepts, slopes and durations with the MAOD method by Medbø et al. (1988) (M0) to calculate the anaerobic contribution during cycling	There were highly significant differences for both the slopes and the intercepts between the different methods (*p* < 0.001); intercepts were significantly different for M4, M6, M7 and M8; slopes were significantly different for M1, M4, M6, M7 and M8; significant differences for the AOD between methods, subjects and durations (*p* < 0.001); overall, M3 showed the best agreement for slope, intercept and between-subject variations
Miyagi et al. (52)	Study A: 14 moderately active males (26 ± 6 years) Study B: 11 mountain bike cyclists (28 ± 4 years)	Study A: 1 graded exercise test with increments of 25 W/2 min until exhaustion; 10 submaximal efforts with 30–80% of VO_2_max; 8 supramaximal efforts at 100–150% of VO_2_max and 70–90 rpm; all tests were performed on a cycle ergometer and on different days Study B: 1 graded exercise test with increments of 25 W/2 min until exhaustion; 2 supramaximal efforts at 115% of VO_2_max; all tests were performed on a cycle ergometer	Comparison of the conventional MAOD and the alternative MAOD (MAOD_ALT_) during different supramaximal intensities on a cycle ergometer (Study A) Investigating the test-retest reliability of MAOD_ALT_ (Study B)	Study A: no significant differences for MAOD and MAOD_ALT_, except for intensities at 130% and 150% of VO_2_max (*p* ≤ 0.048); all MAOD_ALT_ values were moderately significant correlated with MAOD (r = 0.54–0.68; *p* < 0.05); MAOD_ALT_ at 115% VO_2_max showed the highest correlation (r = 0.68; *p* < 0.01); MAOD_ALT_ at 110 and 120% VO_2_max showed highest agreement Study B: no significant differences for MAOD_ALT_ between test and retest (*p* > 0.05); MAOD_ALT_ showed high reproducibility (ICC = 0.81–0.96; *p* < 0.01); significant correlations (r = 0.68–0.96; *p* > 0.05) and good levels of agreement (CV%: 4.1–5.9%) for all values of MAOD_ALT_, except for lactate and phosphagen metabolism
Muniz-Pumares et al. (53)	21 male trained cyclists and triathletes (41 ± 7 years)	1 ramp test (GET) until exhaustion (87 ± 8 rpm); 1 submaximal step test with 10 times 3 min at 50–140% of GET, followed by a ramp test with 70% of GET and increases of 15% of GET every minute until exhaustion; 5 supramaximal tests (105%, 112.5%, 120% and 127.5% of VO_2_max) until exhaustion, lasting ∼2 and 5 min; all tests were separated by at least 48 h	Comparison of AOD at four different supramaximal intensities and investigation of the test-retest reliability of the AOD	ICCs of the AOD and anaerobic contribution were 0.87 and 0.67, respectively; CVs of the AOD and anaerobic contribution were 8.72% and 10.68%, respectively; AOD_112.5_ was significantly higher than AOD_105_ (*p* = 0.033) and AOD_127.5_ (*p* = 0.022); there were no significant differences between AOD_105_, AOD_120_ and AOD_127.5_ (*p* ≥ 0.05); 10% of the participants achieved their MAOD at 105% VO_2_max, 48% at 112.5% VO_2_max, 28% at 120% VO_2_max and 14% at 127.5% VO_2_max, respectively
Noordhof et al. (60)	15 male cyclists (27 ± 6 years)	1 maximal incremental exercise test with intensity increasing 30 W/3 min at pedal frequency of 90 rpm on a cycle ergometer until exhaustion or cadence dropped <80 rpm; 10 exercise bouts of 10 min on a cycle ergometer at intensities of 30–90% of VO_2_max, separated by 20 min rest; 1 pretest lasting 6 min with 60% of VO_2_max, followed by 1 constant-load test at mean power output of a 2.5 km time trial with 90 rpm until pedaling cadence dropped <80 rpm	Comparison of anaerobic contribution calculated with three different MAOD methods (10-Y, 4-Y, 4 + Y) and the GE method during cycling	No significant differences for anaerobic contribution between the four methods (*p* = 0.13); LoA (ml O_2_/kg) were low between the methods: 10-Y vs. GE −3.01 ± 47.2; 4-Y vs. GE −10.4 ± 53.7 and 4 + Y vs. GE −8.87 ± 43.8; there were significant differences for the anaerobic contribution between the methods: 10-Y vs. 4 + Y (*p* < 0.05), 10-Y vs. GE (*p* < 0.01), 4-Y vs. 4 + Y (*p* < 0.001) and 4-Y vs. GE (*p* < 0.01); there was a highly significant main effect for individual anaerobic contribution (*p* < 0.001)
Noordhof et al. (59)	12 male skiers and biathletes (25 ± 3 years) at (inter)national level	Frist day: 12 × 4 min submaximal exercise bouts at different speed (6–24 km/h) and incline levels (2–12%) with roller-skis on a treadmill; 1 maximal incremental exercise test with roller-skis on a treadmill; second day: 21 min simulated mass-start competition with 7 identical laps consisting of 4 segments with different speed and incline with roller-skis on a treadmill and a final all-out sprint	Comparison of 2 MAOD methods (4-Y, 4 + Y) and the GE method to determine the anaerobic energy contribution during XC-skiing (while using different skating sub-techniques)	No significant difference in anaerobic contribution between the 4 methods (*p* = 0.10; w^2^ = 0.08); LoA (kJ) were 5.8 ± 69.1 for GE vs. 4-Y, 28.1 ± 41.2 for GE vs. 4 + Y and 22.3 ± 86.1 for 4-Y vs. 4 + Y; anaerobic contribution was ∼10–15% during the simulated competition
Triska et al. (74)	10 male competitive cyclists (26 ± 4 years)	1 incremental exercise test (GXT) beginning at 40 W and increasing 20 W/min until exhaustion; 3 laboratory tests until exhaustion at 70%, 98%, and 110% of Pmax and a cadence of 100 rpm; 3 field tests with maximal efforts for 2, 6, and 12 min at 85–90 rpm	Comparison of CP and W’ in laboratory and field conditions using 3 different mathematical models (hyperbolic, linear work-time, linear power-1/time)	No significant differences between the 3 mathematical models for CP (*p* = 0.088–1.000) and W’ (*p* = 0.054–0.615) within laboratory and field conditions
Valenzuela et al. (24)	8 males (22 ± 2 years), 12 females (21 ± 1 years) recreationally active in sports	2 incremental exercise tests on a cycle ergometer with increases of 20–30 W every 2 min and a pedaling cadence of 80 rpm until exhaustion or cadence <75 rpm for 5 s; 3 randomized constant power tests on a cycle ergometer with individually selected work rates that lead to exhaustion after ∼4 min and ∼8 min; all tests were separated by at least 48h	Comparison of the anaerobic contribution determined by MAOD or PCr-La-O_2_ method during 4 min and 8 min supramaximal cycling	No significant differences between MAOD and PCr-La-O_2_ for both durations (*p* > 0.05); significantly strong correlations for values of MAOD and PCr-La-O_2_ determined in the 4 min tests (r = 0.93; *p* < 0.01) and in the 8 min tests (r = 0.91; *p* < 0.01); across durations, values were highly correlated between MAOD and PCr-La-O_2_ (r = 0.92; *p* < 0.01); MAOD could be predicted from PCr-La-O_2_ (*p* < 0.01)
Weber and Schneider (54)	7 untrained males (24 ± 1 years) and 7 untrained females (25 ± 2 years)	1 incremental cycling test with intensity increasing 25 W/min for males and 20 W/min for females at 70 rpm until exhaustion; 6 submaximal, randomly ordered 10 min exercise bouts over 2 testing sessions with intensities varying between 20 and 75% of VO_2_peak; 4 supramaximal cycling tests at 110% and 120% of VO_2_peak until exhaustion, randomly ordered and separated by at least 48h	Examination of the test-retest reliability of MAOD determined at 110% and 120% of VO_2_peak during cycling	ICC for MAOD were 0.95 and 0.97 for the 110% and 120% trials (*p* < 0.001); MAOD values were not significantly different between trial 1 and trial 2 for 110% and for 120% (*p* > 0.05); the mean % difference in MAOD between trial 1 and trial 2 was not significantly different for 110% and 120%; the mean MAOD measured for the two 110% trials was not significantly different from the MAOD values obtained from the two 120% trials (2.58 ± 0.18 L vs. 2.64 ± 0.20 L)
Withers et al. (55)	12 subjects (25 ± 5 years) 6 triathletes and 6 cyclists	4 submaximal 10 min tests on a cycle ergometer with power outputs ranging from 103 to 279 W; 4 maximal cycling tests, lasting 45 s, 60 s, 75 s or 90 s	Comparison of MAOD during 45 s, 60 s, 75 s and 90 s of maximal cycling	ICCs for MAOD were highly significant (*p* < 0.001) for 45 s (0.92), 60 s (0.92) and 75 s (0.93); oxygen deficit for the 45 s test was significantly lower than those for 60 s, 75 s, and 90 s (3.52 L vs. 3.75–3.8 L; *p* < 0.01); 3 subjects attained their MAOD during 60 s, 7 subjects during 75 s and 2 subjects during 90 s
Zagatto and Gobatto (64)	9 male table tennis player (18 ± 1 years) at regional and national levels	1 incremental table tennis test with initial intensity of 30 balls/min (∼35 km/h), incremented by 4 balls/2 min until volitional exhaustion; 4 submaximal, 7 min table tennis tests at intensities corresponding to 50%, 60%, 70% and 80% of VO_2_peak; 1 exhaustive table tennis test at 110% of VO_2_peak until exhaustion; 3–4 table tennis tests at intensities between 95 and 130% VO_2_peak until exhaustion (Cf test)	Comparison of W’ derived from three critical power models (linear-f, linear-TB, nonlinear-2) during sub- and supramaximal exercise tests in table tennis Comparison of W’ with MAOD and anaerobic energy contribution (W_ANAER_) during sub- and supramaximal table tennis tests	All W’ values were significantly correlated (ICC = 0.90); none of the W’ values were highly or significantly correlated with MAOD or W_ANAER_ in the Cf test (r = −0.58–0.51; *p* > 0.13); MAOD did not differ significantly from W_ANAER_ in the Cf test (*p* > 0.05); W’ was significantly higher when calculated from nonlinear-2 model than from other models (*p* < 0.05)
Zagatto et al. (56)	Study A: 15 males (24 ± 4 years), moderately active Study B: 14 males (28 ± 5 years), experienced in running	Study A: 1 graded exercise test at 8 km/h with stage increments of 1.5 km/h every 2 min on a treadmill until volitional exhaustion; 10 submaximal efforts at 30–80% of VO_2_max over a 10 min period; 8 supramaximal exercise bouts at 100–150% of VO_2_max until exhaustion, randomized and separated by at least 48h Study B: 1 graded exercise test as performed in Study A; 2 supramaximal efforts with an exercise intensity that resulted in greater concordance and more reliable for MAOD_ALT_ compared to MAOD from Study A	Study A: Comparison of MAOD_ALT_ and conventional MAOD during treadmill running Study B: Assessment of the test-retest reliability of the MAOD_ALT_ method	Study A: MAOD and MAOD_ALT_ values did not differ significantly for absolute (*p* = 0.56) and relative mass (*p* = 0.78); significant correlations were found only for MAOD_ALT_ determined at 100% (r = 0.59; *p* < 0.05) and 115% of VO_2_max (r = 0.73; *p* < 0.05); MAOD_ALT_ at 115% of VO_2_max demonstrated greater concordance based on effect size (−0.12), LoA (−0.08 L ± 0.39) and TE (0.61 L) Study B: ICCs for MAOD_ALT_ were good (0.77–0.87; *p* < 0.001); ICCs for the alactic and lactic contributions were good (ICC = 0.72–0.75, *p* < 0.01); TE for MAOD_ALT_ ranged from 3.52–4.32 ml/kg; LoA for the alactic and lactic contribution were −1.53 ml/kg and 1.26 ml/kg, respectively; mean values for MAOD_ALT_ were not significantly different between test and retest (*p* = 0.85–0.93); lactic and alactic contributions did not differ between test and retest and showed trivial (−0.18) and small (0.45) effect sizes (*p* > 0.05)

ATP, adenosine triphosphate; CCC, concordance correlation coefficient; CP, critical power; CV, coefficient of variation; ES, effect size; GE, gross efficiency; h, hour; ICC, intraclass correlation coefficient; J, joule; kg, kilogram; km/h, kilometers per hour; L, liter; LoA, limits of agreement; m, meter; MAOD, maximal accumulated oxygen deficit; MAP, maximal aerobic power; min, minute, ml, milliliters; mmol, millimole; N, Newton; O_2_ eq/kg oxygen equivalent per kilogram; PCr-La-O_2_, Phosphocreatine-lactate-oxygen; RMSE, root mean square error; rpm, rounds per minute; RQ, respiratory quotient; s, second; SD, standard deviation; SEM, standard error of measurement; TE, typical error; VO_2_, oxygen uptake; VO_2_max, maximum oxygen uptake; VO_2_peak, peak oxygen uptake; W, Watts; W’, anaerobic work capacity; W_ANAER_, anaerobic energy contribution.

In total, 22 studies investigated the reliability and 29 studies investigated the validity of the different methods. Precisely, for the MAOD, 10, 10, 12, and 16 studies evaluated the relative (21, 48–56) and absolute reliability (20, 48–50, 52, 56–60), as well as the criterion (20, 21, 48, 49, 52, 56–58, 61–64) and convergent validity (20, 21, 48, 49, 52, 53, 56–65), respectively. For the PCr-La-O_2_, the relative and absolute reliability were investigated by two studies (12, 66) and three articles reported the criterion and convergent validity (24, 67, 68). For the CP, one study assessed the absolute (64) and relative reliability (69), five (64, 70–73) and seven studies (64, 69–74) reported the criterion and convergent validity, respectively. All quality criteria for the GE were investigated by one study (75). For the bioenergetic model, absolute reliability and criterion and convergent validity were reported by two studies (18, 19).

### 3.2 Quality assessment

Table 3 presents the results of the methodological quality assessment for each method. In total, 22 studies investigated the reliability, of which 4, 14, 1, and 3 were rated as very good, adequate, doubtful, and inadequate quality, respectively. 29 studies assessed the validity with 24, 1, and 4 articles being rated as very good, doubtful, and inadequate quality.

**Table 3:** Results of the methodological quality assessment and best-evidence synthesis.

Method	Criterion	Study (year)	Study Quality	Evidence
MAOD	Reliability	Andersson and McGawley (20)	adequate	strong
		Andersson et al. (57)	very good	
		Andrade et al. (48)	inadequate	
		Bosquet et al. (21)	adequate	
		Bosquet et al. (49)	adequate	
		Campos et al. (58)	doubtful	
		Doherty et al. (50)	adequate	
		Kalva-Filho et al. (51)	adequate	
		Miyagi et al. (52)	adequate	
		Muniz-Pumares et al. (53)	adequate	
		Noordhof et al. (60)	very good	
		Noordhof et al. (59)	very good	
		Weber and Schneider (54)	adequate	
		Withers et al. (55)	adequate	
		Zagatto et al. (56)	adequate	
	Validity	Andersson and McGawley (20)	very good	strong
		Andersson et al. (57)	very good	
		Andrade et al. (48)	very good	
		Bangsbo et al. (61)	inadequate	
		Bosquet et al. (21)	very good	
		Bosquet et al. (49)	very good	
		Buck and McNaughton (62)	very good	
		Campos et al. (58)	very good	
		Hill et al. (68)	very good	
		Medbø and Tabata (7)	very good	
		Medbø and Welde (65)	very good	
		Miyagi et al. (52)	very good	
		Muniz-Pumares et al. (53)	very good	
		Noordhof et al. (60)	very good	
		Noordhof et al. (59)	very good	
		Valenzuela et al. (24)	very good	
		Zagatto and Gobatto (64)	very good	
		Zagatto et al. (56)	very good	
PCr-La-O_2_	Reliability	Kaufmann et al. (12)	inadequate	moderate
		Luches-Pereira et al. (66)	very good	
	Validity	Hatauta et al. (67)	very good	strong
		Hill et al. (68)	very good	
		Valenzuela et al. (24)	very good	
CP	Reliability	Maturana et al. (69)	adequate	limited
		Zagatto and Gobatto (64)	adequate	
	Validity	Bergstrom et al. (70)	inadequate	strong
		Gaesser et al. (71)	very good	
		Hill (72)	doubtful	
		Hill and Smith (73)	very good	
		Maturana et al. (69)	very good	
		Triska et al. (74)	very good	
		Zagatto and Gobatto (64)	very good	
GE	Reliability	Ebreo et al. (75)	inadequate	limited
	Validity	Andersson and McGawley (20)	very good	conflicting
		Andersson et al. (57)	very good	
		Ebreo et al. (75)	very good	
		Noordhof et al. (60)	very good	
		Noordhof et al. (59)	very good	
Bioenergetic model	Reliability	Lidar et al. (18)	adequate	limited
		Lidar et al. (19)	adequate	
	Validity	Lidar et al. (18)	inadequate	limited
		Lidar et al. (19)	inadequate	

For the MAOD, the relative reliability was rated as adequate and inadequate in 9 and 1 studies, respectively. Absolute reliability was very good, adequate, and doubtful for 3, 6, and 1 studies, respectively. Criterion validity was assessed as very good for 12 studies. For the convergent validity, the quality was rated as very good for 15 studies and as inadequate for 1 study. For the PCr-La-O_2_ method, relative and absolute reliability were rated as very good and inadequate, respectively. The criterion and convergent validity were assessed as very good in all 3 studies. For the CP, the relative and absolute reliability were adequate for both studies. The criterion validity was very good, doubtful, and inadequate for 3, 1, and 1 studies, respectively. The quality of the convergent validity was very good, doubtful, and inadequate for 5, 1, and 1 studies, respectively. For GE, reliability was rated as inadequate for one study and validity was very good for the same study. The two studies investigating the reliability for the bioenergetic model were rated as adequate, while the validity was inadequate for two studies.

### 3.3 Synthesis of results

#### 3.3.1 MAOD

The MAOD was the most evaluated method to quantify the aerobic-anaerobic contributions. The reliability and validity were addressed by 15 and 16 studies, respectively. Studies that investigated the reliability mainly used graded exercise tests and several submaximal, constant-load tests with different intensities of maximum oxygen uptake (VO_2_max), or different time trials on a cycle ergometer or treadmill. In two studies, the tests were performed in a swimming pool and during table tennis. Therefore, participants were mainly male runners, cyclists, or recreationally active in sports, but also investigated were swimmers, biathletes and table tennis players. In addition to the conventional MAOD method, an alternative MAOD (MAOD_ALT_) and a backward extrapolation technique were also evaluated (48, 52, 56).

For the MAOD, and in terms of reliability, ICCs were poor to excellent and ranged from 0.26 to 0.97 (21, 49–51, 53–55, 57). CV was excellent between 6.8% and 8.6% and limits of agreement (LoA) ranged from 1.9–6.0% or 15.1-96 ml/kg O_2_ (20, 50, 58). For the MAOD_ALT_, ICC was good to excellent (0.77–0.96), CV was excellent (4.1–5.8%), and TE was low (9.13–12.60 ml/kg) (52, 56). The backward extrapolation technique showed a small ICC (0.26), a poor CV (46.2%), and TE of 24.8 ml/kg O_2_ (48).

In terms of validity, MAOD was evaluated with regard to various calculations, alternative methods and intensities. Zagatto and Gobatto (64) assessed the MAOD and three different CP models during 1 supra- and 4 submaximal tests with various intensities in table tennis. The studies of Bosquet et al. (21, 49) compared different calculations for the MAOD and CP proposed by Medbø (1988), Hill (1998), Morton (1996), Whipp (1986), and Monod and Scherrer (1965). Therefore, they used an incremental test on a treadmill as well as several constant-velocity tests with different intensities in relation to VO_2_max. Results show that the anaerobic contribution was significantly higher when calculated by Morton compared to Whipp or Hill with a large effect (*p* < 0.001; ES = 2.52–2.76) and was largely associated with them (*r* = 0.65–0.75; *p* < 0.05) (21). Additionally, the other study found that there was no significant difference between MAOD values derived from Medbø and Hill (*p* > 0.05) and also, that the small correlation was not statistically significant (*r* = 0.25; *p* > 0.05) (49). The method by Whipp showed largely lower estimations and a large effect for MAOD than the others (bias ± LoA: −29.6 ± 35.6 and −26.1 ± 23.8 ml/kg; *p* < 0.001, ES > 1.94). With regard to potential relations between the methods, correlations were small to moderate, but show no significant association between MAOD from Hill with other estimates (*r* = 0.21–0.33; *p* > 0.05) and no significant relationship between Whipp and Medbø (*r* = 0.33; *p* > 0.05) (49). In table tennis, the comparisons of the MAOD and CP models were neither large nor significant (*r* = 0.06–0.16; *p* > 0.05) (64). The anaerobic contribution calculated by three different MAOD calculations and the GE was compared by four studies (20, 57, 59, 60). Therefore, they included or excluded the y-intercept as a baseline metabolic rate and used 4- or 10-minutes submaximal exercise bouts for running or roller-skiing on a treadmill or for cycling on an ergometer. In two studies, Noordhof et al. (59, 60) found no significant differences between the four (10-Y, 4-Y, 4 + Y, GE) methods in cycling (*p* = 0.13) and skiing (*p* = 0.10). Furthermore, LoA between the methods were 10-Y vs. GE −3.01 ± 47.2 ml O_2_/kg, 4-Y vs. GE −10.4 ± 53.7 ml O_2_/kg, and 4 + Y vs. GE −8.87 ± 43.8 ml O_2_/kg. In contrast, Andersson et al. (57) found significantly lower estimations of anaerobic contribution by the 5 + Y_LIN_ method compared to the three other models (∼26%; *p* = 0.002). In the fourth study, the oxygen deficit was significantly lower with 4 + Y compared to 4-Y and GE/EC (ES = 0.64; *p* < 0.05) (20). The mean difference (bias) between the oxygen deficit estimated with the 4 + Y vs. 4-Y method was −6.3 ± 4.9 ml/kg, with the 4 + Y vs. GE/EC method −7.2 ± 1.2 ml/kg, and with the 4-Y vs. GE/EC method −1.0 ± 5.3 ml/kg, respectively. With regard to correlations, the oxygen deficits estimated with the 4 + Y vs. GE/EC method were highly and significantly correlated (*r* = 0.99; *p* < 0.05) (20). In another study, anaerobic contribution determined by conventional MAOD method and backward extrapolation technique was compared at different submaximal running intensities (48). No significant differences were found between the conventional MAOD values and backward technique values (*p* > 0.05). Additionally, a large correlation between conventional MAOD and backward extrapolation for absolute (r = 0.86) and relative (r = 0.85) MAOD was demonstrated (48). The MAOD was compared to an alternative model (MAOD_ALT_) in two studies during different cycling and running intensities (52, 56). Both studies could not ascertain significant differences for MAOD and MAOD_ALT_, except for intensities at 130% and 150% of VO_2_max (*p* ≤ 0.048). Moreover, all MAOD_ALT_ values were largely significant correlated with MAOD (*r* = 0.54–0.68; *p* < 0.05), but Zagatto et al. (56) only found significant correlations at 100% (r = 0.49–0.59; *p* < 0.05) and 115% (r = 0.65–0.77; *p* < 0.05) of VO_2_max. With regard to intensities, MAOD_ALT_ demonstrated the largest correlation with MAOD (*r* = 0.68; *p* < 0.01) and the greatest concordance at 115% VO_2_max (*r* = 0.73; *p* < 0.01) (52, 56). For the conventional MAOD, Muniz-Pumares et al. (53) showed that AOD at 112.5% of VO_2_max was significantly greater than AOD at 105% (*p* = 0.033) and AOD at 127.5% (*p* = 0.022) during cycling. There were no significant differences (*p* ≥ 0.05) between AOD at 105, 120, and 127.5% VO_2_max. There were two studies that compared the oxygen deficit with muscle biopsies (61, 63). They concluded that they relate extremely well and that the ATP turnover rate determined from the oxygen deficit or from muscle biopsies are similar (91.2 vs. 91.6 mmol ATP/kg wet weight). Three studies investigated the relationship between the MAOD and PCr-La-O_2_ method (24, 67, 68). Recreationally active males and females as well as runners performed several constant-power tests with different intensities and at least one incremental test on a cycle ergometer (24, 67) or a treadmill (68). The correlation for PCr-La-O_2_ and MAOD was very small and not significant (*r* = −0.06; *p* > 0.05), but also no significant difference was found between the calculated anaerobic contribution from PCr-La-O_2_ and MAOD method (44.6 ± 3.0% vs. 45.2 ± 5.1%; *p* = 0.79) (67). In contrast, the other two studies demonstrated largely significant correlations with a small to moderate effect between MAOD and PCr-La-O_2_ for every test duration and across test durations (*r* = 0.80–0.99; ES = 0.32–0.52; *p* ≤ 0.01) (24, 68). However, there was no interaction effect, but MAOD could be predicted from PCr-La-O_2_ (*p* ≤ 0.01) (24, 68).

#### 3.3.2 PCr-La-O_2_

The PCr-La-O_2_ method was evaluated by two studies investigating the reliability and three studies addressing the validity. Recreationally active males and females as well as male state-level handball players participated in the reliability studies. The testing protocols involved either a knee-extensor exercise test at 100% and 110% of peak power or an intermittent running test. For the intermittent running test, both the PCr-La-O₂ model and the intermittent PCr-La-O₂ model were analyzed. In general, reliability was stronger for the 100% test than for the 110% test. ICC was moderate (0.71; *p* = 0.004) and poor (0.44; *p* = 0.085), CVs were excellent to poor (3.3–60.4%) and LoA were between 753.5 to −591.7 ml O₂ and 1,002.4 to −1,188.0 ml O₂ for the test at 100% and at 110%, respectively. Additionally, the standard error of measurement (SEM) ranged from 240.1 ml O₂ to 389.6 ml O₂ (66). For the conventional and intermittent PCr-La-O₂ model, the highest variability was found for the alactic contribution of the conventional (CV = 14.85%) and intermittent (CV = 9.98%) model. The overall anaerobic contribution showed low variability and excellent CVs for the conventional (CV = 7.49%) and intermittent model (CV = 8.95%). LoA varied across energy contributions, with the widest range observed for the anaerobic contribution of the intermittent model (−1,448 to 664 J/kg). The alactic contributions also showed notable variability, with LoA ranges of −368 to 439 J/kg and −1,707 to 988 J/kg, respectively (12).

The criterion and convergent validity were investigated in three studies, with AOD and MAOD being the comparators (24, 67, 68). A detailed discussion of the results was provided in the previous section.

#### 3.3.3 CP

Concerning the CP, two and seven studies examined the reliability and validity of the method, respectively. One study assessed the absolute reliability during table tennis and compared the anaerobic contribution derived from three different critical power models (linear-f, linear-TB, nonlinear-2) (64). The second study used an incremental ramp test and five constant-power tests on a cycle ergometer to assess the relative reliability of CP. Therefore, CP and W' were estimated by five different mathematical models (CP_exponential_, CP3-_hyperbolic_, CP2-_hyperbolic_, CP_linear_, and CP_1/time_) and five different numbers of time to exhaustion trials, resulting in 34 different combinations (69). Agreement for all W' values was good to excellent in both studies (ICC = 0.90, CCC = 0.78–0.99). Root mean square error (RMSE) ranged from 2.44 to 22.90 W and was lowest for CP_linear (2,3,4,5)_ and highest for CP_1/time (1,2)_. The model that predicted data most accurately was the CP3-_hyp(1,2,3,4,5)_, (R^2^ = 0.99, RMSE = 26.5 W).

In total, seven studies investigated the validity of CP. Mainly recreationally trained males, females and cyclists were included, but also table tennis players took part. The participant cohorts primarily included recreationally trained males, females, and cyclists, with table tennis players also included in one study. All tests were conducted on a cycle ergometer using both incremental and constant-intensity protocols, except for the study of Zagatto and Gobatto (64), which utilized supra- and submaximal table tennis-specific tests. Gaesser et al. (71) compared the anaerobic contribution estimated by five different CP models [3-parameter nonlinear, 2-parameter nonlinear, linear (P x t), linear (P), exponential] during cycling. Significant differences (*p* > 0.05) were observed between the models, with the three-parameter nonlinear model yielding the highest anaerobic contribution (58 ± 19 kJ) and the linear (P) model the lowest (18 ± 5 kJ). Additionally, the goodness of fit was significantly lower for the linear (P) model (R^2^ = 0.96 ± 0.03; *p* = 0.005) compared to all other models. Large correlations were found between anaerobic contribution estimates from the two linear models (*r* = 0.97; *p* < 0.001) and the two-parameter nonlinear model (*r* = 0.96–0.99; *p* < 0.001), whereas strong to small correlations were observed for the three-parameter nonlinear model (*r* = 0.25–0.64; *p* > 0.05-*p* < 0.01). With regard to other models, there was a significantly large correlation between the linear power-time relationship and the MAOD (r = 0.77; *p* < 0.01) (73). However, none of the W’ values were highly or significantly correlated with MAOD, anaerobic alactic (W_PCr_), anaerobic alactic energy contribution (W_La_) or anaerobic energy contribution (W_ANAER_) (r = 0.06–0.60; *p* > 0.05) during table tennis (64). Five studies investigated the convergent validity of CP. Therefore, different mathematical calculations (linear, nonlinear, hyperbolic, exponential) were compared. Bergstrom et al. (70) demonstrated highly significant differences between linear-TW, linear-P, nonlinear-2, nonlinear-3, and CP_3min_ model (*p* < 0.001). Additionally, nonlinear-3 and nonlinear-2 models produced significantly higher estimates of anaerobic contributions than the linear-TW, linear-P and CP models (*p* < 0.05). The same result was shown by Gaesser et al. (71). Anaerobic contribution estimates differed significantly between the five models, of which the 3-parameter model provided the highest and the linear (P) model the lowest anaerobic contribution (58 ± 19 kJ vs. 18 ± 5 kJ; *p* < 0.008). Similarly, Hill (72) demonstrated that CP was highest when derived from the 3-parameter exponential model (209 ± 51 W), with significant differences observed among the three models (2-parameter model, 3-parameter hyperbolic model, 3-parameter exponential model) (*p* = 0.003). Precisely, anaerobic contribution was significantly higher when derived from the 3-parameter compared to the 2-parameter hyperbolic model (25.3 ± 13.2 vs. 20.4 ± 9.0 kJ; *p* = 0.048). However, in table tennis, W' was significantly higher when calculated from nonlinear-2 model compared to other models (linear-f, linear-TB, nonlinear-2) (*p* < 0.05) (64). In contrast to these findings, Triska et al. (74) demonstrated no significant differences for CP (*p* = 0.088–1.000) and W' (*p* = 0.054–0.615) between hyperbolic, linear work-time, and linear power-1/time models during cycling within laboratory or field conditions. Regarding the influence of model selection and exercise durations, one study observed that W′ was overestimated when derived from CP_linear_- and CP_1/time_-model, particularly in trials lasting less than 10 minutes. Conversely, trials of approximately 20 minutes provided the most accurate estimation of W' (69).

#### 3.3.4 GE

One study investigated the absolute and relative reliability as well as the criterion and convergent validity (75). Males and females with a minimum of six hours training per week performed one incremental cycling test and a cycling test with intensities of 50% and 80% or 100% of maximal aerobic power twice. The aim was to compare the anaerobic contribution between the conventional GE method and the backward extrapolation GE method (BGE). Mean CVs were excellent (7.8% and 9.8%) for BGE. For the anaerobic contribution, CVs were also excellent (3.5% vs. 2.9% and 6.8% vs. 5.0% for GE vs. BGE). LoA for GE vs. BGE were 3.6% vs. ±3.74% and ±4.2% vs. ±4.1% (75).

With regard to validity, GE and BGE demonstrated highly significant and large correlations after the first (*r* = 0.98; *p* = 0.01) and second trial (*r* = 0.80; *p* = 0.01), indicating high agreement between methods. Further, the GE was compared to different MAOD models (10-Y, 4-Y, 4 + Y, 5 + Y_LIN_) in four studies (20, 57, 59, 60). They used 4-, 5- or 10-minutes submaximal exercise bouts for cycling on an ergometer as well as for running or roller-skiing on a treadmill. In two studies, there were no significant differences between the MAOD and GE methods in cycling (*p* = 0.13) and skiing (*p* = 0.10; w^2^ = 0.08) (59, 60). Additionally, LoA between MAOD and GE were between −10.4 and 53.7 ml O_2_/kg. Contrary, anaerobic contribution was significantly lower when estimated by a MAOD model (5 + Y_LIN_) compared to GE (*p* = 0.002) (57). Similar results were demonstrated by Andersson and McGawley (20), where the oxygen deficit was significantly lower with 4 + Y compared to 4-Y and GE/EC (ES = 0.64; *p* < 0.05). The mean difference between the oxygen deficit estimated with the 4 + Y vs. GE/EC method was −7.2 ± 1.2 ml/kg and with the 4-Y vs. GE/EC method −1.0 ± 5.3 ml/kg. Moreover, the oxygen deficits estimated with the 4 + Y vs. GE/EC method were highly correlated (*r* = 0.99; *p* < 0.05) (20).

#### 3.3.5 Bioenergetic model

Two studies invented and evaluated the bioenergetic model (18, 19). In the first study, 11 male cross-country skiers at national and international level performed 4 submaximal exercise tests and 2 self-paced roller-skiing sprint time trials (STT) on a treadmill. The aim was to compare four bioenergetic models (2TM-fixed, 2TM-free, 3TM-fixed and 3TM-free) estimating the aerobic and anaerobic contribution during sprint roller-skiing (18). For the second study, 14 well-trained cyclists performed one submaximal incremental cycling test, one maximal incremental cycling test, and two intermittent protocols with various power outputs to compare the measured and modelled metabolic energy supply (19).

The model-to-measurement mean difference (0.5) and TE of the anaerobic contribution were lower but not significant for the 2TM-free compared to the other models (TE = 0.6; *p* = 0.103). Additionally, the RMSE of anaerobic contribution were the lowest for the 2TM-free and the highest for the 3TM-fixed model (11.7% vs. 17.2%; 50.0–77.6 W vs. 104.1–106.1 W) (18). With regard to measured data, the RMSE for the aerobic contribution was 61.9 ± 7.9 W with LoA ranging from −124.8 W to 119.2 W (19).

Concerning the validity, over- and underprediction were highest by the 3TM-free model and by the 3TM-fixed model, respectively. The relative contribution from the alactic and lactic system to the total anaerobic contribution was 38.6% and 61.4% for the 3TM-free and 38.7% and 61.3% for the 3TM-fixed model, respectively (18). Furthermore, the modelled aerobic contribution shows a small underprediction compared to the measured aerobic contribution (8.6 ± 1.5%). In addition, there were significant differences (*p* ≤ 0.001–0.036) between modelled and measured data at several different stages during the intermittent protocol (19).

### 3.4 Best-evidence synthesis

Table 3 shows the result of the best-evidence synthesis, structured according to the different methods. For the MAOD, evidence of reliability was rated as strong based on 15 studies with very good (*n* = 3), adequate (*n* = 10), doubtful (*n* = 1), and inadequate (*n* = 1) study quality. Of the 16 studies assessing the validity, 15 studies were rated as very good and one study as inadequate, leading to overall strong evidence. Concerning the PCr-La-O_2_, there was moderate evidence for the reliability due to one study of very good quality and one of inadequate. Evidence for validity was strong based on three high-quality studies. In terms of the CP, two studies of adequate quality led to limited evidence for reliability. In contrast, evidence of validity was strong due to 5, 1, and 1 studies of very good, doubtful, and inadequate quality, respectively. Concerning the GE, one study of inadequate and very good quality led to limited and moderate evidence of reliability and validity, respectively. The evidence for the bioenergetic model was limited based on two studies of adequate quality for reliability and inadequate quality for validity.

## 4 Discussion

The aim of this systematic review was to assess the reliability and validity of different methods used to quantify the aerobic-anaerobic energy contributions during sports and exercise, and thereby clarify the level of evidence for each method. The main findings regarding reliability and validity were: (i) evidence was strong for MAOD, (ii) evidence was limited to strong for CP and PCr-La-O_2_, and (iii) evidence was limited to conflicting for GE and the bioenergetic model.

To our knowledge, this is the first systematic review to implement a best-evidence synthesis for this topic, aiming to establish an overview of the methodological quality and empirical support for each method. As expected, MAOD was clearly the most extensively investigated method. In general, MAOD emerged as the most evaluated method and the only one with strong evidence for both reliability and validity. In contrast, the reliability of CP, PCr-La-O₂, and GE has been minimally investigated, each with only two studies, resulting in at most moderate evidence. Reliability was generally less investigated than validity and was evaluated using stricter criteria, particularly concerning participant's stability, protocol consistency, and statistical analyses. However, reliability is essential for understanding measurement error and ensuring accurate interpretation of performance changes. Therefore, further research is warranted to clarify the reliability of the different methods used to quantify aerobic-anaerobic energy contributions during sports and exercise.

The first main finding of this study was that evidence was strong for MAOD in terms of reliability and validity. Among all evaluated methods, MAOD demonstrated the strongest evidence for reliability, supported by 15 studies of adequate to very good quality as well as consistent findings (Table 3). Except for the backward extrapolation technique, high ICCs along with low CVs and LoA indicate the method's robustness in repeated measurements. Thus, MAOD is a reliable method for quantifying the oxygen deficit and anaerobic contribution. The evidence for the validity of MAOD is equally supported. A notable strength of MAOD lies in its consistent methodological evaluation across multiple studies, the majority of which were rated as having very good study quality (Table 3). However, the results of the studies investigating the validity demonstrate that there are a few methodological aspects to consider when applying the MAOD. For instance, MAOD is highly sensitive to protocol configurations due to its dependence on accurate estimations of both aerobic demand and actual oxygen uptake. Several investigations have demonstrated that variables such as intensity, duration, and slope calculations used to construct the VO₂-exercise intensity relationship directly affect the reliability of the estimated oxygen demand (53, 62, 65). The standard protocol typically involves ten submaximal 10-minute bouts to generate a robust VO₂-power output regression, presenting a high physiological and logistical burden. Even small deviations in these parameters can alter the linearity assumption or affect steady-state conditions, thereby distorting the aerobic-anaerobic energy balance calculated by MAOD (60, 62). Another important aspect is the specificity of the exercise modality. While MAOD has been primarily assessed in controlled settings like treadmill or cycle ergometry, its extension to sport-specific or variable-intensity environments is limited. The only study to apply MAOD in a sport-specific context was conducted in tethered swimming (58). The requirement for constant intensity and steady-state conditions makes it difficult to apply in sports characterized by intermittent or technical movements. Since the MAOD is a two-component model only, it does not differentiate between anaerobic lactic and alactic energy contributions. Consequently, this may limit its interpretative value for performance diagnostics and resulting training recommendations. Nevertheless, MAOD offers the most reliable and valid framework for estimating anaerobic energy contributions among currently available methods.

The second main finding was that evidence was limited to strong for the CP and PCr-La-O_2_ concerning the reliability and validity (Table 3). Current evidence for the reliability of CP remains limited. Although two studies in cycling and table tennis reported good to excellent ICCs or CCCs, the small number and only adequate quality investigations limit the strength of this evidence. Notably, test protocols involving efforts under 10 minutes were associated with lower CCCs and a tendency to over- or underestimate CP, emphasizing the model's sensitivity to test duration. Since the CP model assumes a linear relationship between work and time above CP, this assumption only holds true within a specific time domain, typically between 2 and 15 minutes. Trials that are too short (<2 minutes) tend to overestimate anaerobic capacity and inflate W′, while longer trials (>20 minutes) may underestimate CP due to factors like fatigue, motivation, or pacing (69). Inconsistent or poorly distributed trial durations can lead to inaccurate curve fitting, distorting both CP and W′ estimates. In contrast, evidence supporting CP's validity is strong, based on five high-quality studies (Table 3). Findings consistently showed that hyperbolic or exponential models yield higher anaerobic estimates than linear ones, with three-parameter models outperforming two-parameter models in both accuracy and robustness (69–72). Unlike MAOD, CP requires fewer submaximal trials and therefore, reducing the methodological and participant's burden. However, the relationship between CP and MAOD is inconsistent. Hill and Smith (73) found significant correlations, Zagatto and Gobatto (64) did not, potentially due to differences in exercise modalities (cycling vs. table tennis). This shows that although CP has been widely validated in cycling, it shows reduced generalizability to sport-specific exercise. Similar to MAOD, CP is also a two-component model only that is not able to distinguish between anaerobic alactic and lactic energy contributions. However, the PCr-La-O_2_ is a three-component model and is currently the only method that is able to separate the anaerobic energy contribution into lactic and alactic share. This distinction is especially valuable because it enables direct quantification of anaerobic alactic (via EPOC_fast_) and lactic (via lactate accumulation) components. Since the alactic energy contribution is calculated from the fast component of the EPOC, it could only be assessed right after the end of an exercise. However, an intermittent PCr-La-O_2_ method was developed, which considers the aerobic phosphocreatine restoration during short breaks (76). Despite this strength, the current evidence for the reliability is moderate, based on two studies of contrasting quality. Both studies reported small CVs and moderate to excellent ICCs, supporting the method's overall reliability (12, 66). However, the anaerobic alactic and lactic components were found to be less reliable than the aerobic component, particularly in intermittent exercise protocols (12). This may be attributed to the method's dependence on VO₂ off-kinetics, which introduces variability when estimating the fast component of EPOC and therefore, affects the quantification of the alactic contribution. However, in terms of validity, evidence is strong. All three validation studies were of high quality and reported generally consistent findings (Table 3). Importantly, the PCr-La-O_2_ method is independent of submaximal pretests or threshold-based models and appeared less sensitive to exercise duration than MAOD, suggesting its robustness across short-duration efforts. Moreover, the method demonstrated consistent performance across both cycling and running protocols, and no sex differences were identified, supporting its broader applicability, but smaller evidence compared to MAOD. The methodological advantages and validation in multiple sports make it a promising tool for quantifying the anaerobic energy contribution validly in both laboratory and field-based settings, requiring more research and development.

The last main finding was that evidence was limited to conflicting for GE and the bioenergetic model. The reliability of GE has been evaluated in a single study (75), which was rated as inadequate, therefore resulting in overall limited evidence (Table 3). Nevertheless, the study reported excellent CVs and narrow LoA for the backward extrapolation technique during cycling, suggesting that this specific variant of GE may offer promising reliability. In terms of validity, the evidence for GE remains conflicting (Table 3). The inconsistencies regarding the comparison with MAOD likely stem from differences in protocols and calculations. GE typically uses only one submaximal exercise bout, thus offering a more practical and time-efficient solution. However, this simplicity may compromise accuracy, possibly leading to an overestimation of anaerobic energy contributions. Supporting this, the BGE method yielded higher anaerobic estimates than conventional GE, but its strong correlations and favorable reliability metrics suggest it could be a viable alternative. The bioenergetic model has similarly limited empirical support, with reliability and validity assessed in only two studies (18, 19), rated as adequate and inadequate, respectively (Table 3). It is based on a three-component energy system framework and includes a highly detailed parameterization of metabolic pathways. While this complexity allows for detailed modelling and the distinction between anaerobic alactic and lactic share, it may also favor measurement error. In particular, TE and RMSE were lowest for the two-component model compared to three-component models (Lidar et al., 2021). Importantly, while the model shows excellent agreement with measured aerobic metabolism, its estimations of anaerobic contribution remain inadequately validated. Both GE and the bioenergetic model demonstrate limited and inconsistent evidence, with some promising features, but overall lack sufficient validation for accurately assessing the anaerobic energy contribution.

## 5 Conclusion

To quantify aerobic-anaerobic energy contributions during sports and exercise, the MAOD has emerged as the most evaluated method and the only one with strong evidence for both reliability and validity. However, as the PCr-La-O_2_ method is the only approach that can distinguish between anaerobic alactic and lactic contributions using direct physiological measures, it should be further evaluated.

## Data Availability

The original contributions presented in the study are included in the article/Supplementary Material, further inquiries can be directed to the corresponding author.
