# Supplementary material for: Evaluation of methods to quantify aerobic-anaerobic energy contributions during sports and exercise — a systematic review and best-evidence synthesis
Source: Front Sports Act Living. 2025 Sep 25;7:1650741. doi: 10.3389/fspor.2025.1650741 (PMC12507823; doi:10.3389/fspor.2025.1650741)
Supplement: Supplementary file 1 [file Datasheet1.docx]

Assessment of methodological quality based on the boxes 6 – 9a of the COSMIN checklist

|  | Andersson et al. 2021 | Andersson&McGawley, 2018 | Andrade et al. 2021 | Bangsbo et al. 1990 | Bergstrom et al. 2014 | Bosquet et al. 2007 | Bosquet et al. 2008 | Buck & McNaughton 1999 | Campos et al. 2022 | Doherty et al. 2000 |  |  |
| --- | --- | --- | --- | --- | --- | --- | --- | --- | --- | --- | --- | --- |
| **Box 6. Reliability** | | | |  |  |  |  |  |  |  |  |  |
| Design requirements | | | |  |  |  |  |  |  |  |  |  |
| 1. Were patients stable in the interim period on the construct to be measured? | NA | NA | 2 | NA | NA | 2 | 2 | NA | NA | 2 |  |  |
| 2. Was the time interval appropriate? | NA | NA | 3 | NA | NA | 3 | 3 | NA | NA | 3 |  |  |
| 3. Were the test conditions similar for the measurements? e.g. type of administration, environment, instructions | NA | NA | 3 | NA | NA | 3 | 3 | NA | NA | 3 |  |  |
| Statistical methods | | | |  |  |  |  |  |  |  |  |  |
| 4. For continuous scores: Was an intraclass correlation coefficient (ICC) calculated? | NA | NA | 2 | NA | NA | 3 | 2 | NA | NA | 3 |  |  |
| Other | | | |  |  |  |  |  |  |  |  |  |
| 8. Were there any other important flaws in the design or statistical methods of the study? | NA | NA | 3 | NA | NA | 3 | 3 | NA | NA | 3 |  |  |
| **Box 7. Measurement error** |  |  |  |  |  |  |  |  |  |  |  |  |
| Design requirements | | | |  |  |  |  |  |  |  |  |  |
| 1. Were patients stable in the interim period on the construct to be measured? | 2 | 3 | 2 | NA | NA | NA | 2 | NA | 2 | 2 |  |  |
| 2. Was the time interval appropriate? | 3 | 3 | 3 | NA | NA | NA | 3 | NA | 3 | 3 |  |  |
| 3. Were the test conditions similar for the measurements? (e.g. type of administration, environment, instructions) | 3 | 3 | 3 | NA | NA | NA | 3 | NA | 3 | 3 |  |  |
| Statistical methods | | | |  |  |  |  |  |  |  |  |  |
| 4. For continuous scores: Was the Standard Error of Measurement (SEM), Smallest Detectable Change (SDC) or Limits of Agreement (LoA) calculated? | 3 | 3 | 0 | NA | NA | NA | 3 | NA | 3 | 3 |  |  |
| Other | | | |  |  |  |  |  |  |  |  |  |
| 6. Were there any other important flaws in the design or statistical methods of the study? | 3 | 3 | 3 | NA | NA | NA | 3 | NA | 1 | 3 |  |  |
| **Box 8. Criterion validity** | | | |  |  |  |  |  |  |  |  |  |
| Statistical methods | | | |  |  |  |  |  |  |  |  |  |
| 1. For continuous scores: Were correlations, or the area under the receiver operating curve calculated? | 3 | 3 | 3 | 0 | 0 | 3 | 3 | 3 | 3 | NA |  |  |
| Other | | | |  |  |  |  |  |  |  |  |  |
| 3. Were there any other important flaws in the design or statistical methods of the study? | 3 | 3 | 3 | 3 | 3 | 3 | 3 | 3 | 3 | NA |  |  |
| **Box 9a. Convergent validity** | | | |  |  |  |  |  |  |  |  |  |
| Design requirements | | | |  |  |  |  |  |  |  |  |  |
| 1 Is it clear what the comparator instrument(s) measure(s)? | 3 | 3 | 3 | 3 | 3 | 3 | 3 | 3 | 3 | NA |  |  |
| 2 Were the measurement properties of the comparator instrument(s) sufficient? | 3 | 3 | 3 | 3 | 3 | 3 | 3 | 3 | 3 | NA |  |  |
| Statistical methods | | | |  |  |  |  |  |  |  |  |  |
| 3 Were design and statistical methods adequate for the hypotheses to be tested? | 3 | 3 | 3 | 2 | 3 | 3 | 3 | 3 | 3 | NA |  |  |

3 = very good; 2 = adequate; 1 = doubtful; 0 = inadequate, and NA = not applicable.

|  | Ebreo et al. 2019 | Gaesser et al. 1995 | Hatauta et al. 2024 | Hill 2004 | Hill et al. 2020 | Hill & Smith 1993 | Kalva-Filho et al. 2016 | Kaufmann et al. 2022 | Lidar et al. 2023 |  |  |  |
| --- | --- | --- | --- | --- | --- | --- | --- | --- | --- | --- | --- | --- |
| **Box 6. Reliability** | | | |  |  |  |  |  |  |  |  |  |
| Design requirements | | | |  |  |  |  |  |  |  |  |  |
| 1. Were patients stable in the interim period on the construct to be measured? | 1 | NA | NA | NA | NA | NA | 2 | 2 | NA |  |  |  |
| 2. Was the time interval appropriate? | 1 | NA | NA | NA | NA | NA | 3 | 3 | NA |  |  |  |
| 3. Were the test conditions similar for the measurements? e.g. type of administration, environment, instructions | 2 | NA | NA | NA | NA | NA | 3 | 3 | NA |  |  |  |
| Statistical methods | | | |  |  |  |  |  |  |  |  |  |
| 4. For continuous scores: Was an intraclass correlation coefficient (ICC) calculated? | 0 | NA | NA | NA | NA | NA | 2 | 0 | NA |  |  |  |
| Other | | | |  |  |  |  |  |  |  |  |  |
| 8. Were there any other important flaws in the design or statistical methods of the study? | 3 | NA | NA | NA | NA | NA | 3 | 3 | NA |  |  |  |
| **Box 7. Measurement error** |  |  |  |  |  |  |  |  |  |  |  |  |
| Design requirements | | | |  |  |  |  |  |  |  |  |  |
| 1. Were patients stable in the interim period on the construct to be measured? | 1 | NA | NA | NA | NA | NA | NA | 2 | 2 |  |  |  |
| 2. Was the time interval appropriate? | 1 | NA | NA | NA | NA | NA | NA | 3 | 3 |  |  |  |
| 3. Were the test conditions similar for the measurements? (e.g. type of administration, environment, instructions) | 2 | NA | NA | NA | NA | NA | NA | 3 | 3 |  |  |  |
| Statistical methods | | | |  |  |  |  |  |  |  |  |  |
| 4. For continuous scores: Was the Standard Error of Measurement (SEM), Smallest Detectable Change (SDC) or Limits of Agreement (LoA) calculated? | 3 | NA | NA | NA | NA | NA | NA | 3 | 3 |  |  |  |
| Other | | | |  |  |  |  |  |  |  |  |  |
| 6. Were there any other important flaws in the design or statistical methods of the study? | 3 | NA | NA | NA | NA | NA | NA | 3 | 3 |  |  |  |
| **Box 8. Criterion validity** | | | |  |  |  |  |  |  |  |  |  |
| Statistical methods | | | |  |  |  |  |  |  |  |  |  |
| 1. For continuous scores: Were correlations, or the area under the receiver operating curve calculated? | 3 | 3 | 3 | 3 | 3 | 3 | NA | NA | 0 |  |  |  |
| Other | | | |  |  |  |  |  |  |  |  |  |
| 3. Were there any other important flaws in the design or statistical methods of the study? | 3 | 3 | 3 | 1 | 3 | 3 | NA | NA | 3 |  |  |  |
| **Box 9a. Convergent validity** | | | |  |  |  |  |  |  |  |  |  |
| Design requirements | | | |  |  |  |  |  |  |  |  |  |
| 1 Is it clear what the comparator instrument(s) measure(s)? | 3 | 3 | 3 | 3 | 3 | 3 | NA | NA | 3 |  |  |  |
| 2 Were the measurement properties of the comparator instrument(s) sufficient? | 3 | 3 | 3 | 3 | 3 | 3 | NA | NA | 3 |  |  |  |
| Statistical methods | | | |  |  |  |  |  |  |  |  |  |
| 3 Were design and statistical methods adequate for the hypotheses to be tested? | 3 | 3 | 3 | 3 | 3 | 3 | NA | NA | 3 |  |  |  |

3 = very good; 2 = adequate; 1 = doubtful; 0 = inadequate, and NA = not applicable.

|  | Lidar et al. 2021 | Luches-Pereira et al. 2024 | Maturana et al. 2018 | Medbo & Tabata 1993 | Medbo & Wede 2022 | Miyagi et al. 2017 | Muniz-Pumares et al. 2017 | Noordhof et al. 2011 | Noordhof et al. 2021 |  |  |  |  |
| --- | --- | --- | --- | --- | --- | --- | --- | --- | --- | --- | --- | --- | --- |
| **Box 6. Reliability** | | | |  |  |  |  |  |  |  |  |  |  |
| Design requirements | | | |  |  |  |  |  |  |  |  |  |  |
| 1. Were patients stable in the interim period on the construct to be measured? | NA | 3 | NA | NA | NA | 2 | 2 | NA | NA |  |  |  |  |
| 2. Was the time interval appropriate? | NA | 3 | NA | NA | NA | 3 | 3 | NA | NA |  |  |  |  |
| 3. Were the test conditions similar for the measurements? e.g. type of administration, environment, instructions | NA | 3 | NA | NA | NA | 3 | 3 | NA | NA |  |  |  |  |
| Statistical methods | | | |  |  |  |  |  |  |  |  |  |  |
| 4. For continuous scores: Was an intraclass correlation coefficient (ICC) calculated? | NA | 3 | NA | NA | NA | 3 | 3 | NA | NA |  |  |  |  |
| Other | | | |  |  |  |  |  |  |  |  |  |  |
| 8. Were there any other important flaws in the design or statistical methods of the study? | NA | 3 | NA | NA | NA | 3 | 3 | NA | NA |  |  |  |  |
| **Box 7. Measurement error** |  |  |  |  |  |  |  |  |  |  |  |  |  |
| Design requirements | | | |  |  |  |  |  |  |  |  |  |  |
| 1. Were patients stable in the interim period on the construct to be measured? | 2 | 3 | 2 | NA | NA | 2 | NA | 3 | 3 |  |  |  |  |
| 2. Was the time interval appropriate? | 3 | 3 | 3 | NA | NA | 3 | NA | 3 | 3 |  |  |  |  |
| 3. Were the test conditions similar for the measurements? (e.g. type of administration, environment, instructions) | 3 | 3 | 3 | NA | NA | 3 | NA | 3 | 3 |  |  |  |  |
| Statistical methods | | | |  |  |  |  |  |  |  |  |  |  |
| 4. For continuous scores: Was the Standard Error of Measurement (SEM), Smallest Detectable Change (SDC) or Limits of Agreement (LoA) calculated? | 3 | 3 | 2 | NA | NA | 3 | NA | 3 | 3 |  |  |  |  |
| Other | | | |  |  |  |  |  |  |  |  |  |  |
| 6. Were there any other important flaws in the design or statistical methods of the study? | 3 | 3 | 3 | NA | NA | 3 | NA | 3 | 3 |  |  |  |  |
| **Box 8. Criterion validity** | | | |  |  |  |  |  |  |  |  |  |  |
| Statistical methods | | | |  |  |  |  |  |  |  |  |  |  |
| 1. For continuous scores: Were correlations, or the area under the receiver operating curve calculated? | 0 | NA | NA | 3 | NA | 3 | NA | NA | NA |  |  |  |  |
| Other | | | |  |  |  |  |  |  |  |  |  |  |
| 3. Were there any other important flaws in the design or statistical methods of the study? | 3 | NA | NA | 3 | NA | 3 | NA | NA | NA |  |  |  |  |
| **Box 9a. Convergent validity** | | | |  |  |  |  |  |  |  |  |  |  |
| Design requirements | | | |  |  |  |  |  |  |  |  |  |  |
| 1 Is it clear what the comparator instrument(s) measure(s)? | 3 | NA | 3 | 3 | 3 | 3 | 3 | 3 | 3 |  |  |  |  |
| 2 Were the measurement properties of the comparator instrument(s) sufficient? | 3 | NA | 3 | 3 | 3 | 3 | 3 | 3 | 3 |  |  |  |  |
| Statistical methods | | | |  |  |  |  |  |  |  |  |  |  |
| 3 Were design and statistical methods adequate for the hypotheses to be tested? | 3 | NA | 3 | 3 | 3 | 3 | 3 | 3 | 3 |  |  |  |  |

3 = very good; 2 = adequate; 1 = doubtful; 0 = inadequate, and NA = not applicable.

|  | Triska et al. 2015 | Valenzuela et al. 2020 | Weber & Schneider 2001 | Withers et al. 1993 | Zagatto et al. 2016 | Zagatto & Gobatto 2012 |  |  |  |  |
| --- | --- | --- | --- | --- | --- | --- | --- | --- | --- | --- |
| **Box 6. Reliability** | | | |  |  |  |  |  |  |  |
| Design requirements | | | |  |  |  |  |  |  |  |
| 1. Were patients stable in the interim period on the construct to be measured? | NA | NA | 2 | 2 | 3 | 2 |  |  |  |  |
| 2. Was the time interval appropriate? | NA | NA | 3 | 1 | 3 | 3 |  |  |  |  |
| 3. Were the test conditions similar for the measurements? e.g. type of administration, environment, instructions | NA | NA | 2 | 3 | 3 | 3 |  |  |  |  |
| Statistical methods | | | |  |  |  |  |  |  |  |
| 4. For continuous scores: Was an intraclass correlation coefficient (ICC) calculated? | NA | NA | 2 | 2 | 2 | 3 |  |  |  |  |
| Other | | | |  |  |  |  |  |  |  |
| 8. Were there any other important flaws in the design or statistical methods of the study? | NA | NA | 3 | 3 | 3 | 3 |  |  |  |  |
| **Box 7. Measurement error** |  |  |  |  |  |  |  |  |  |  |
| Design requirements | | | |  |  |  |  |  |  |  |
| 1. Were patients stable in the interim period on the construct to be measured? | NA | 2 | NA | NA | 3 | NA |  |  |  |  |
| 2. Was the time interval appropriate? | NA | 3 | NA | NA | 3 | NA |  |  |  |  |
| 3. Were the test conditions similar for the measurements? (e.g. type of administration, environment, instructions) | NA | 3 | NA | NA | 3 | NA |  |  |  |  |
| Statistical methods | | | |  |  |  |  |  |  |  |
| 4. For continuous scores: Was the Standard Error of Measurement (SEM), Smallest Detectable Change (SDC) or Limits of Agreement (LoA) calculated? | NA | 2 | NA | NA | 3 | NA |  |  |  |  |
| Other | | | |  |  |  |  |  |  |  |
| 6. Were there any other important flaws in the design or statistical methods of the study? | NA | 3 | NA | NA | 3 | NA |  |  |  |  |
| **Box 8. Criterion validity** | | | |  |  |  |  |  |  |  |
| Statistical methods | | | |  |  |  |  |  |  |  |
| 1. For continuous scores: Were correlations, or the area under the receiver operating curve calculated? | NA | 3 | NA | NA | 3 | 3 |  |  |  |  |
| Other | | | |  |  |  |  |  |  |  |
| 3. Were there any other important flaws in the design or statistical methods of the study? | NA | 3 | NA | NA | 3 | 3 |  |  |  |  |
| **Box 9a. Convergent validity** | | | |  |  |  |  |  |  |  |
| Design requirements | | | |  |  |  |  |  |  |  |
| 1 Is it clear what the comparator instrument(s) measure(s)? | 3 | 3 | NA | NA | 3 | 3 |  |  |  |  |
| 2 Were the measurement properties of the comparator instrument(s) sufficient? | 3 | 3 | NA | NA | 3 | 3 |  |  |  |  |
| Statistical methods | | | |  |  |  |  |  |  |  |
| 3 Were design and statistical methods adequate for the hypotheses to be tested? | 3 | 3 | NA | NA | 3 | 3 |  |  |  |  |

3 = very good; 2 = adequate; 1 = doubtful; 0 = inadequate, and NA = not applicable.
